# Isolation, Genomic Analysis, and Preliminary Application of a Bovine *Klebsiella pneumoniae* Bacteriophage vB_Kpn_B01

**DOI:** 10.3389/fvets.2021.622049

**Published:** 2021-09-03

**Authors:** Zidan Luo, Shangjingchao Geng, Biao Lu, Guangli Han, Yin Wang, Yan Luo, Zexiao Yang, Suizhong Cao, Xueping Yao

**Affiliations:** ^1^College of Veterinary Medicine, Sichuan Agricultural University, Chengdu, China; ^2^Key Laboratory of Animal Disease and Human Health of Sichuan Province, Chengdu, China

**Keywords:** *Klebsiella pneumoniae*, bacteriophage, biological properties, whole-genome sequencing, preliminary treatment test

## Abstract

*Klebsiella pneumoniae* is an important pathogen that can infect both humans and cattle. The widespread *K. pneumoniae* and its high drug resistance make it difficult to treat Klebsiella infections/diseases. In this study, a lytic *K. pneumoniae* bacteriophage vB_Kpn_B01 was isolated from a dairy farm trough in Sichuan Province, and its biological properties were studied, and the entire genome of vB_Kpn_B01 was sequenced. The therapeutic effects of the phage on disease-causing mice were preliminarily tested. Phages found in this study are double-stranded DNA bacterial viruses belonging to the *family Siphoviridae, Sugarlandvirus*. The results suggest that vB_Kpn_B01 has strong specificity and low adaptability to different adverse conditions. Meanwhile, the predicted gene products of phage vB_Kpn_B01 comprised 149 coding sequences (CDS) and 25 tRNAs, of which 34 CDS had known functions. Of course, vB_Kpn_B01 did not contain any known antibiotic-resistant or virulent genes. The pathological sections of the liver and lungs of mice showed that the inflammatory scores of the treatment group were lower than in the bacterial group. Phage vB_Kpn_B01 alleviated the inflammatory response in the organs of the infected mice, and the organ tissue bacterial load of the treatment group was significantly lower than that of the bacterial group. Therefore, vB_Kpn_B01 can inhibit the proliferation of *K. pneumoniae* 18 *in vivo* and can alleviate the inflammation of target organs caused by infectious bacteria, which preliminarily indicates that vB_Kpn_B01 has a certain therapeutic effect on laboratory-infected mice.

## Introduction

*Klebsiella pneumoniae* (*K. pneumoniae*) is a gram-negative bacterium that belongs to the genus, *Klebsiella* of *Enterobacteriaceae*, which is widely distributed in nature and is a common conditional pathogen (1). It mainly exists in the intestines and respiratory tracts of humans and animals. *K. pneumoniae* is a conditional pathogen associated with a wide spectrum of infections, such as pneumonia, hysteritis, and sepsis (2). When the body's immune function is reduced or the overuse of antibiotics leads to bacterial flora imbalance, *K. pneumoniae* infection can cause several diseases and even death (3). In animal husbandry, *K. pneumoniae* causes severe pneumonia, sepsis, meningitis, and mastitis in cattle (4). Mastitis caused by *Klebsiella* infection is often more severe (5, 6). Relevant reports in China also indicate that *K. pneumoniae* is a pathogenic bacterium that is highly pathogenic to bovine (7–9). In recent years, the abuse of antibacterial drugs has led to the widespread resistance of *K. pneumoniae*, which has resulted in great difficulty in treating Klebsiella infections/diseases. In 2001, Saidel-Odes et al. (10) reported on the existence of carbapenem-resistant *K. pneumoniae* in France. Since then, many countries around the world have discovered the existence of drug-resistant strains (11–13). This has resulted in great difficulties in treating Klebsiella infections/disease and has attracted a high degree of clinical attention.

Many epidemiological surveys have shown that in recent years, due to the abuse of various antibacterial drugs, the resistance of *K. pneumoniae* is widespread, and most of the isolates are multi-drug resistant strains, which have brought some difficulty in treating Klebsiella infections/diseases. Given the increasing number of multi-drug resistant *K. pneumoniae*, scientists are exploring the use of bacteriophages to replace antibiotics. There are two types of bacteriophages: temperate bacteriophages, which do not proliferate or lyse host bacteria under normal conditions; and virulent bacteriophages, which directly replicate and lyse the bacteria after entering the host cell, with more suitability to replace antibiotics in clinical use (14). At present, the specific virulent bacteriophage of *Streptococcus pneumoniae* has been proven to control infections caused by the pathogen (15), and research on the elimination of *S. pneumoniae* biofilms by lytic phages has also made effective progress (16). Many clinical research studies have shown that, bacteriophages not only exert good control of bacterial infections but are also harmless to the body and have almost no side effects (17, 18). Considering the strong specificity and antibacterial ability of phages, research on the prevention and control of bacterial diseases by phages has attracted much attention worldwide (19).

Here, we isolated a *K. pneumoniae* bacteriophage, vB_Kpn_B01, from a dairy farm in Sichuan Province, China. The general biological properties of vB_Kpn_B01 were determined, and the genome characteristics were analyzed, and a mouse infection model was established to demonstrate the therapeutic efficacy, which could provide a scientific basis for the application of *K. pneumoniae* phage.

## Methods

### Bacterial Strains and Cultivation Media

The strain used in this study was maintained in the Animal Quarantine Laboratory of Sichuan Agricultural University. Bacterial strains were identified by sequencing a 1,308-bp fragment of the 16S rRNA gene (20). SM buffer, LB broth, LB agar plates (1.2% w/v), and LB soft agar (0.6% w/v) were used to cultivate the phage and its host strains.

### Phage Isolation and Purification

Bacteriophage vB_Kpn_B01 was isolated from a water trough obtained from a dairy farm in Sichuan Province, China. Bacteriophage isolation and propagation were performed as previously described (21) with appropriate modifications. Water samples were incubated overnight at 37°C in LB broth and then centrifuged at 8,000 × g for 10 min. This supernatant was filtered through a 0.22 μm Millipore filter and checked for the presence of phages. One hundred microliters of filtrate and 100 μl indicator bacteria were added to 2 ml of warm soft agar (0.6% w/v), mixed, and poured on Petri dishes (1.2% w/v) to form plaques. The plates were incubated at 37°C for 12 h and plaque formation was observed. Phages were purified by picking a single plaque with a sterile Pasteur pipette tip, resuspended in 1 ml SM buffer, static culturing overnight at 4°C, and then using the double-agar layer method. Phage purification was repeated 3–5 times until the plaques formed the same. Purified phages were amplified and stored at −70 °C.

### Transmission Electron Microscopy (TEM)

Purified phages were dropped on a copper mesh covered with carbon film and allowed to stand for 15 min, and the excess liquid on the copper mesh was blotted with filter paper. Then, 2% phosphotungstic acid (PTA, pH = 7.0) was added dropwise for 10 min, dried naturally, and phages were observed using a transmission electron microscope (Lanzhou Veterinary Research Institute, Lanzhou, China).

### Thermal and pH Tests

Phage suspensions of titer (10^9^ pfu/ml) were incubated at 40, 50, 60, 70, and 80°C for 20, 40, and 60 min, respectively. The phages (10^9^ pfu/ml) were incubated in suspension buffers at pH 3, 5, 9, and 11. All samples were collected at 37°C for 1 h and the residual phage titer was assayed by the double-layer agar method.

### Multiplicity of Infection (MOI) Assay and One-Step Growth Curve Assay

We took 1 ml *K. pneumoniae* 18, which grew in the logarithmic phase, added the phage to the multiplicity of infection of 0.01, 0.1, 1, 10, and 100, and cultured at 37°C for 6 h. Then, 100 μl of the culture medium was added and diluted gradually. The double-agar layer method was used to determine the phage titer. The test was repeated thrice to obtain the optimal multiplicity of infection.

Bacteriophages (10^9^ pfu/ml) and indicator bacteria were mixed with an optimal multiplicity of infection, incubated at 37°C, and taken at 20, 40, 60, 90, 120, and 160 min. The phage titer was determined using the double-agar layer method. The latent period, burst time, and burst size were calculated from the one-step growth curves.

### DNA Extraction and Whole-Genome Sequence

Phage DNA was extracted and purified from the phage lysates using the Viral RNA/DNA Extraction Kit (TaKaRa MiniBEST Viral RNA/DNA Extraction Kit Ver.5.0). The results were tested by 0.8 % agarose gel electrophoresis.

Next-generation sequencing (NGS) was used to sequence the complete genome of bacteriophage vB_Kpn_B01 with a MiSeq PE300 sequencer (Shanghai Tp Biotechnology Co., LTD, Shanghai, China). After obtaining the sequencing data, the whole phage genome sequence was assembled using Newbler2.9. The coding sequences (CDSs) in the genome were predicted using Prokka (22). The annotation sequences were verified using the NCBI Microbial Genome Submission Check. Phage genome annotation was visualized using the CGView Server (http://stothard.afns.ualberta.ca/cgview_server/). Phylogenetic analysis of the proteins was performed using MEGA 6.0, using the neighbor-joining method. The annotated genome sequence of vB_Kpn_B01 was deposited in GenBank under the accession number MT380195.1.

### Phage Treatment Test

Altogether, 24 mice were randomly divided into four groups randomly (6 for each group). As shown in Table 1, group A corresponds to the control, group B corresponds to the bacteria, group C corresponds to the phage, and group D corresponds to bacteria+phage (Treat). The intraperitoneal injection dose of all 4 groups was 0.2 mL. Mice were fed and observed for mental status at 48 h; thereafter, mice were anesthetized using 5% chloral hydrate (0.1 mL/10 g) and sacrificed. The lungs and livers were removed, fixed in 4% formaldehyde, and embedded in paraffin. The 5-μm-thick tissues were sliced and stained with hematoxylin and eosin. The degree of inflammation was determined using the Ishak (23) standard score. The liver and lung samples were homogenized in sterile saline, and the homogenized tissue samples were diluted and plated onto nutrient agar and cultured at 37°C for 24 h to evaluate the bacterial load.

**Table 1:** Injection of mice in each group.

**Groups**	**SM Buffer**	**Bacteria (KP 18)**	**Phage (vB_Kpn_B01)**
A (Control)	+	–	–
B (Bacteria)	–	+	–
C (Phage)	–	–	+
D (Treat)	–	+	+

*+, injection; –, without injection*.

Statistical analysis was performed using GraphPad Prism software (version 8.0). The data shown in the tables were compared for significance using analysis of variance (ANOVA). Statistical significance was set at *p* <0.05.

## Results

### Isolation and Morphology

**Figure 1:**
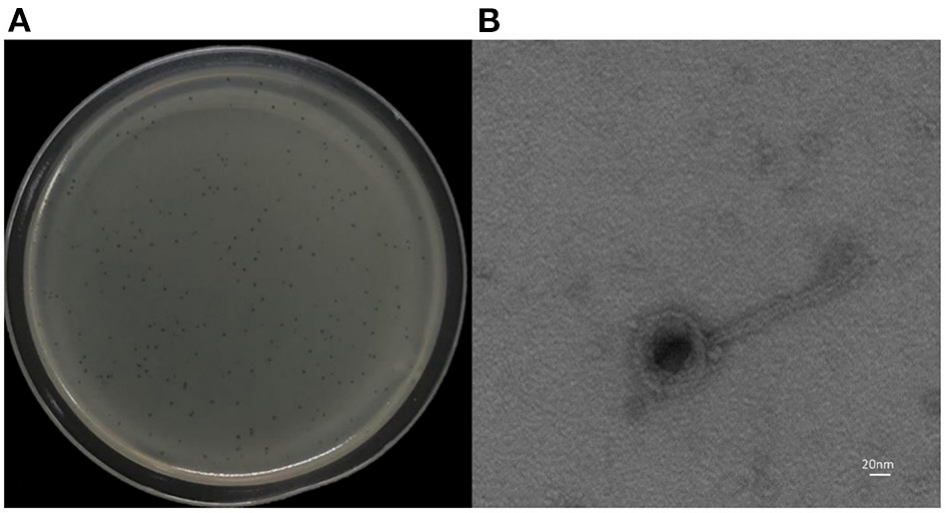
Morphology of phage vB_Kpn_B01. **(A)** Plaques of the phage; **(B)** transmission electron micrograph of the phage.

*K. pneumoniae* 18 was used as a host strain for the isolation of the bacteriophage vB_Kpn_B01. The vB_Kpn_B01 phage formed clear plaques with clear boundaries. Each plaque ranged from 1.5 to 2 mm (Figure 1A). Transmission electron microscopy revealed phage capsids with a diameter of 60–80 nm, and a tail with a length of approximately 160 nm, a width of approximately 10 nm, a short tail approximately 16 nm in length (Figure 1B). The phage belongs to the family *Siphoviridae*.

### Thermal and pH Tests

**Figure 2:**
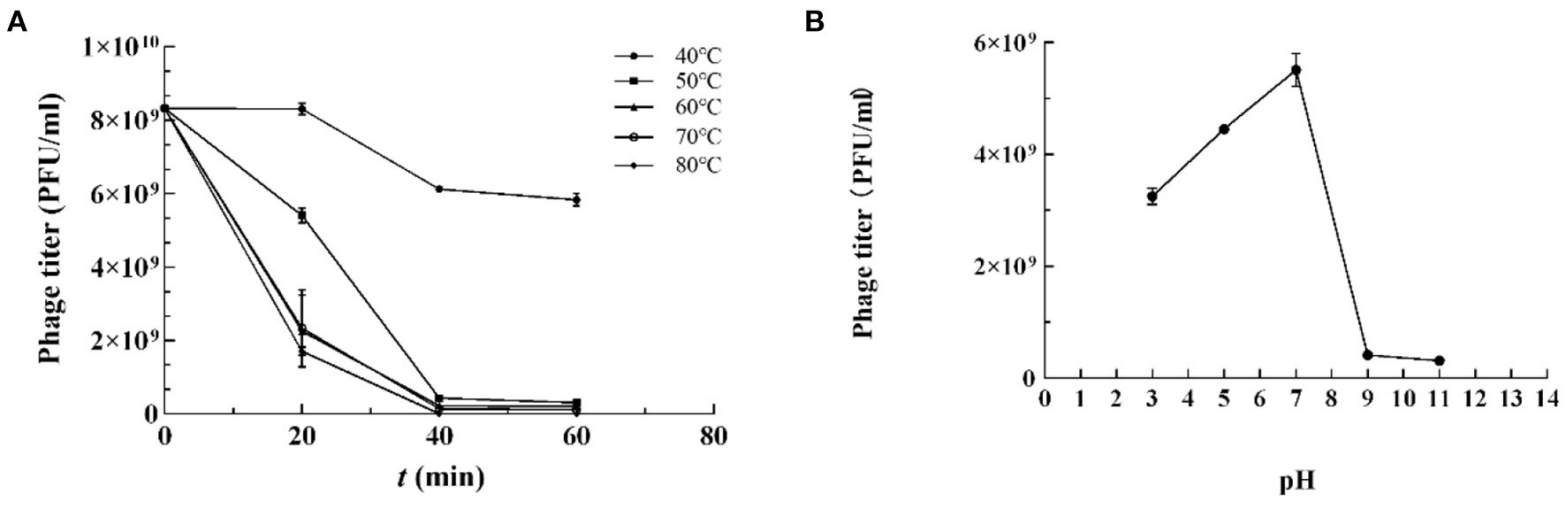
Resistance of *K. pneumoniae* phage vB_Kpn_B01 to temperature. **(A)** Resistance of phage to temperature; **(B)** Resistance of phage pH.

The stability of vB_Kpn_B01 was investigated under different thermal and pH conditions based on the input and residual plaque-forming unit (pfu) numbers. The study revealed that phages tend to survive in a wide range of temperatures with a decline in counts at and above 50°C. However, the phage was completely inactivated when heated to 80°C for 40 min (Figure 2A).

*K. pneumoniae* phage vB_Kpn_B01 was found to be stable in a pH range of 4–7, retained lytic capabilities when incubated for 1 h at 37°C at pH 4–7. However, extreme pH (<4 or > 9) PFU counts declined substantially (Figure 2B).

### MOI and One-Step Growth Curve Assay

vB_Kpn_B01 had the highest titer at MOI = 0.01, which is the best multiplicity of phage infection (Table 2).

**Table 2:** Determination of optimal multiplicity of infection (MOI) of vB_Kpn_B01.

**MOI**	**Bacteria/CFU**	**Phage/PFU**	**Phage titer/(PFU/mL)**
0.01	1 × 10^9^	1 × 10^7^	8.9 × 10^11^
0.1	1 × 10^9^	1 × 10^8^	8.00 × 10^10^
1	1 × 10^9^	1 × 10^9^	3.94 × 10^10^
10	1 × 10^8^	1 × 10^9^	3.02 × 10^10^
100	1 × 10^7^	1 × 10^9^	4.19 × 10^10^

**Figure 3:**
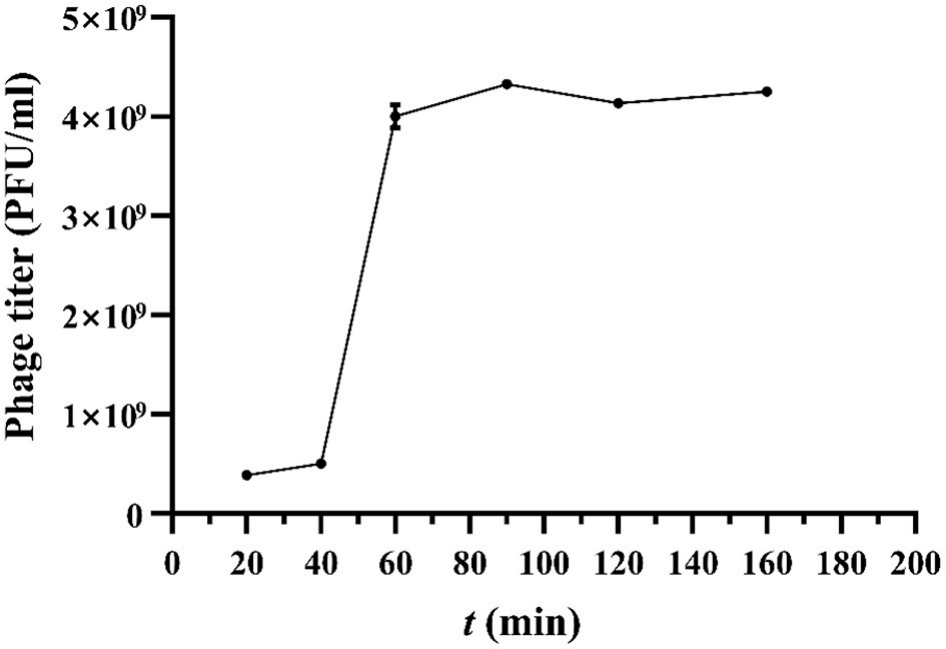
One-step growth curve of vB_Kpn_B01.

According to the one-step growth curve (Figure 3), the incubation period of phage was 40 min, and the lysis period was 60 min to 90 min. The burst size for vB_Kpn_B01 was 40±3 PFU/infected cells. The burst size was calculated as the ratio of the final count of liberated phage particles to the initial count of infected bacterial cells.

### Genome Analysis of vB_Kpn_B01

The size of the vB_Kpn_B01 genome was 113,227 bp, and the GC content of the phage genome was 47.97%. The BLASTn results showed that the whole-genome of vB_Kpn_B01 was most similar to that of vB_Kpn_IME260(GenBank: NC_041899.1), with 97% genome coverage and 97.38% sequence identity.

### Predicted Gene Products

**Figure 4:**
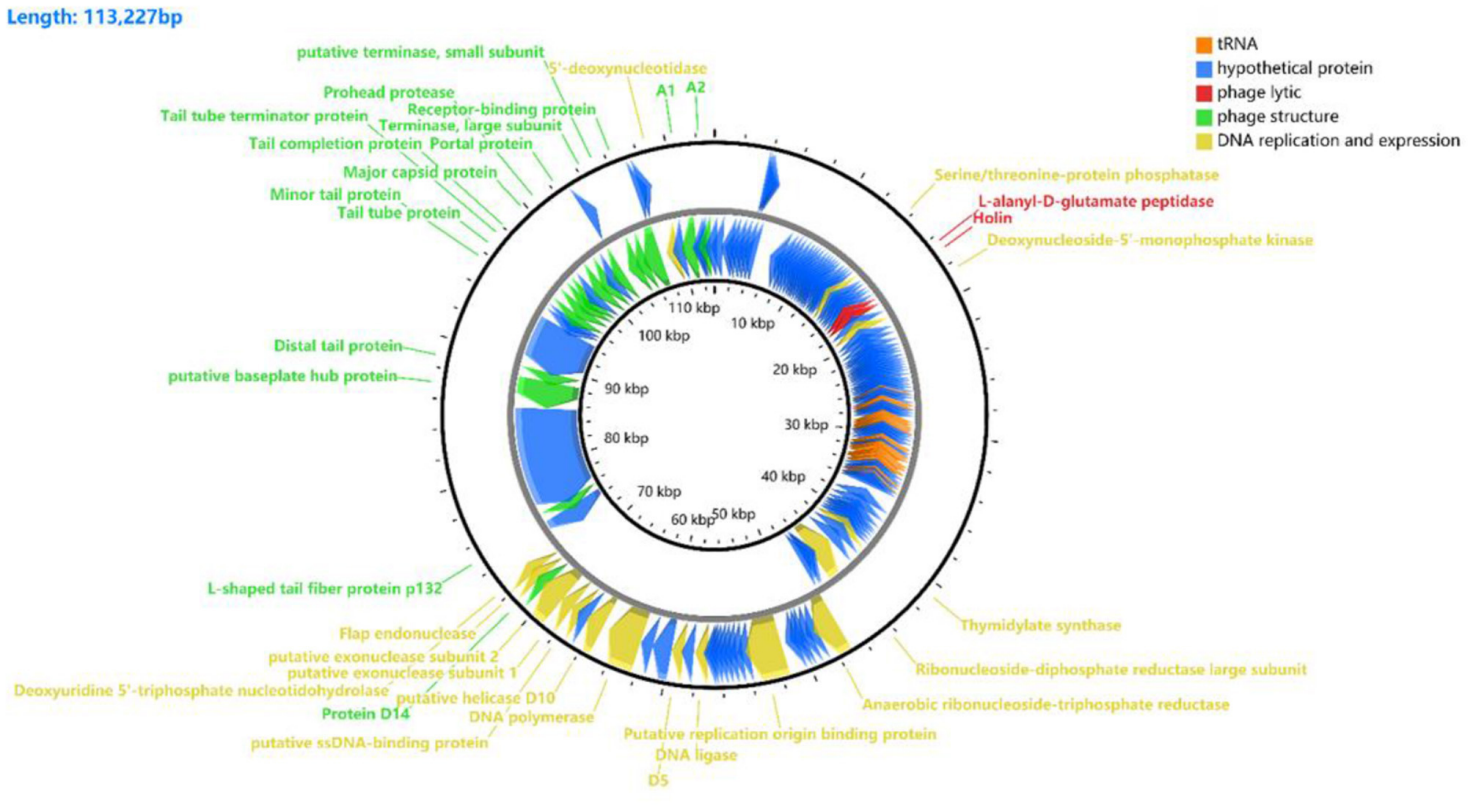
Gene functional annotation of vB_Kpn_B01. The arrow represents the direction of the gene, and the color represents different domains of functional genes: red, lyase-related genes; orange, tRNA; yellow, DNA replication and gene expression genes; green, structural protein genes; blue, hypothetical protein genes.

Results from the Prokka predicted 149 coding sequences (CDS) and 25 tRNAs, of which 34 CDS had known functions, and 115 CDS (an additional table shows this in more detail (see Supplementary Table 1). Among these CDSs, 35 were transcribed in a forward direction, while the other genes were transcribed in reverse. The whole-genome includes lyase-related genes (red), tRNAs (orange), DNA replication and expression genes (yellow), structural protein genes (green), and hypothetical protein genes (blue) (Figure 4).

Functional analysis of the phage CDS showed that the genes of the phage form a modular structure, including the modules that encode the structural gene cluster (16 CDS), DNA replication and gene expression cluster (16 CDS), and gene cluster associated with host cell lysis (2 CS).

Among the 34 genes with putative functions, 16 were annotated as structural protein-encoding genes, including the head (gp159, gp163, gp164), tail (gp146, gp148, gp149, gp153, gp154, gp155, gp156), and others (gp142, gp158, gp161, gp165, gp170, and gp173). The structural protein-encoding genes were located close together in the genome, which might benefit the regulation of the phage's life cycle after infecting the host bacterium.

Genomic annotation of the phage revealed that 16 of the phage genes were associated with DNA replication and gene expression, including gp30, gp39, gp109, gp112, gp116, gp122, gp131, gp133, gp136, gp137, gp139, gp140, gp141, gp143, gp144, and gp168. These proteins are involved in DNA metabolism, repair, recombination, auxiliary metabolism, and gene expression. The different transcription directions and locations of these genes might be due to the different expression times of these genes after infection.

Two genes were annotated as cell lyase-related genes, gp36 (L-alanyl-D-glutamate peptidase) and gp37 (Holin). These genes can help the phage lyse the bacterium to release its progeny virions in the later stages of phage reproduction. No genes were found to produce and transmit virulence factors in the vB_Kpn_B01 phages by genomic functional annotation.

### Phylogenetic Analysis of vB_Kpn_B01

Genes encoding proteins such as the major capsid protein are conserved and can be used as signature genes for the classification of phages.

**Figure 5:**
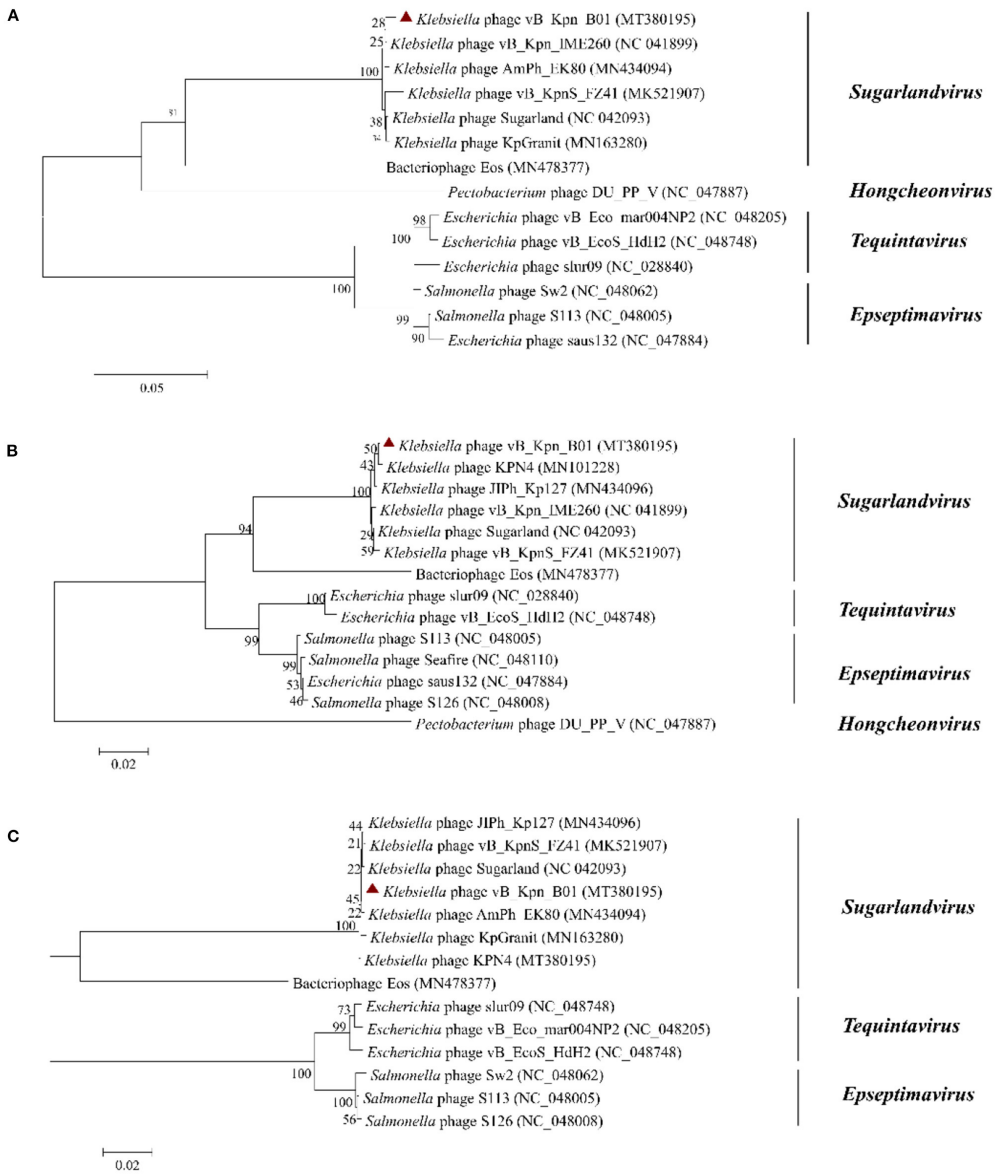
Phylogenetics trees were constructed based on major capsid protein **(A)**, terminase large subunit **(B)**, and DNA polymerase **(C)** of the phage. The number on the branch point represents credibility (the closer the value is to 100, the stronger the credibility). The length of the branches represents the genetic distance; the shorter the ruler, the closer the relationship. Red triangle: phage vB_Kpn_B01.

To further analyze the genetic relationship between vB_Kpn_B01 and other phages of *the Siphoviridae* family, we used conserved and evolutionally significant amino acid sequences of the major capsid protein (gp158), terminal enzyme large subunit (gp163), and DNA polymerase (gp136) to construct a genetic evolutionary tree (Figure 5) (24, 25). As shown in the figure, vB_Kpn_B01 belongs to *Caudovirales, Siphoviridae, Sugarlandvirus*.

### Phage Treatment Test

After injection of SM Buffer, KP 18, or vB_Kpn_B01 mice were observed daily for clinical symptoms. Mice in the bacterial challenge group (Group B) were mentally depressed within 24 h of the challenge, feed intake was decreased, aspired due to chills, and within 2 days, eyes were red and swollen with a few purulent secretions. After the challenge in the treatment group (Group D), psychosis and dietary status of mice were observed, with the injection of phage (4 h after challenge) normalizing appetite within 2 days, but mental status inactive compared to the blank control group.

**Figure 6:**
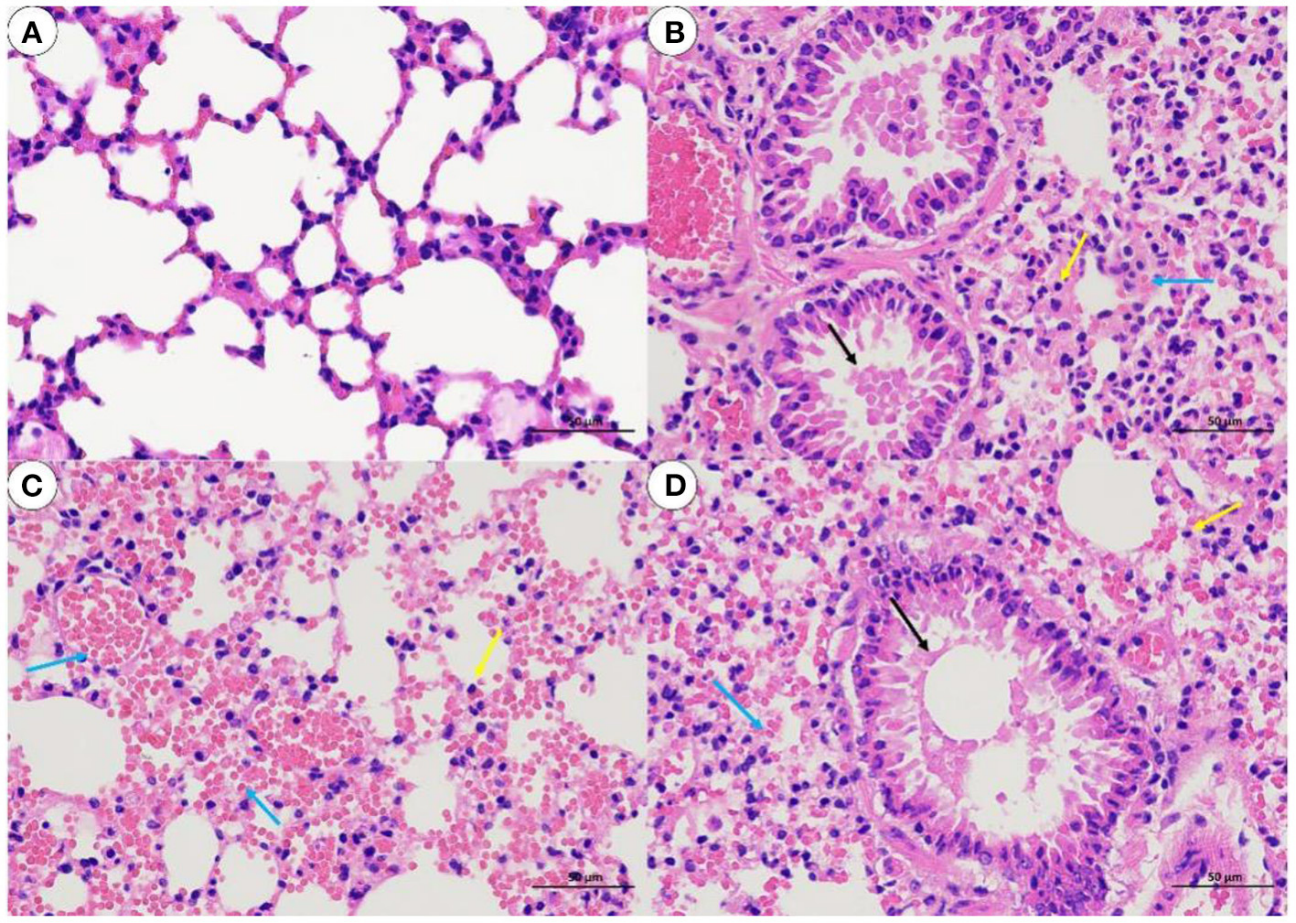
Lung sections from mice (H. E, 400 X). **(A)** blank control group; **(B)** bacterial challenge group; **(C)** phage group; **(D)** treatment group; inflammatory cells (yellow arrow); airway luminal secretions (black arrows); bleeding or congestion (blue arrow).

**Figure 7:**
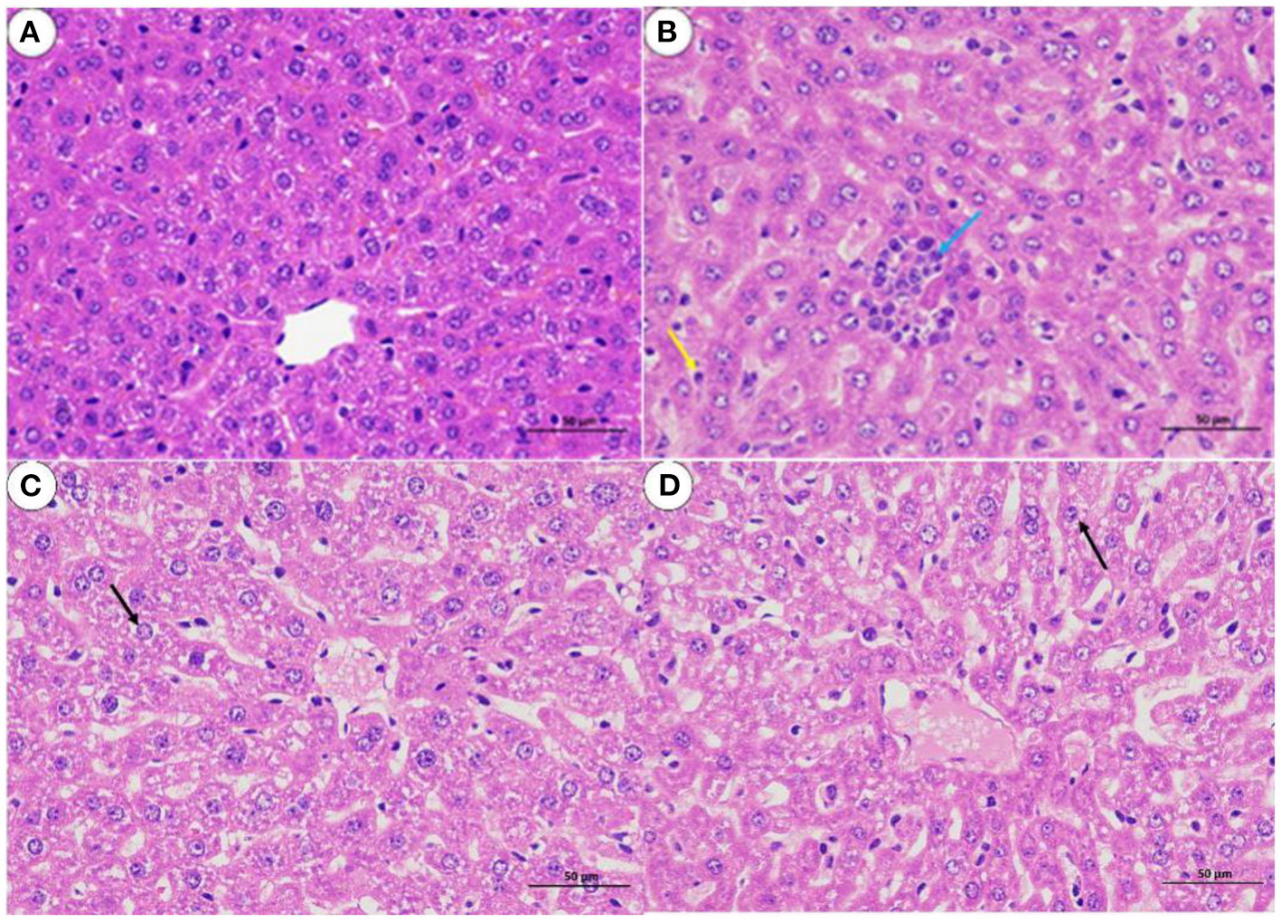
Liver sections from mice (H. E, 400 X). **(A)** blank control group; **(B)** bacterial challenge group; **(C)** phage group; **(D)** treatment group; inflammatory cells (blue arrow); white blood cells (yellow arrow); particle degeneration (black arrow).

The histological analysis results were as follows: For lung samples, the bacterial group (Figure 6B) and treatment group (Figure 6D) exhibited congestion accompanied by the infiltration of inflammatory cells. Combined with the lung lesion scores (Table 3), the inflammatory scores of the treatment group were lower than those of the bacterial group. For liver samples, compared with the blank control group (Figure 7A), the treatment group (Figure 7D) had a clear liver tissue structure, and no obvious inflammatory cell infiltration was observed. However, in the bacterial group (Figure 7B), the number of leukocytes in the hepatic sinusoids increased, and a few foci of inflammatory cell infiltration were observed in the hepatic lobules. Liver inflammatory scores were slightly higher in the bacterial group than in the treatment group (Table 4).

**Table 3:** Grading of histopathological in the lungs of mice.

**Group**	**Inflammatory**	**Thickening**	**Congestion**	**Hemorrhage**	**Airway luminal secretions**
A	0	0	0	0	0
B	2	2	3	0	4
C	1	0	4	2	0
D	1	0	4	1	1

*No lesions or very few lesions were scored as 0; slight lesions were scored as 1; moderate lesions were scored as 2; severe lesions were scored as 3; very severe lesions were scored as 4*.

**Table 4:** Grading of histopathological in the livers of mice.

**Group**	**Denaturation**	**Inflammatory**
A	0	0
B	0	1
C	1	0
D	1	0

*No lesions or very few lesions were scored as 0; slight lesions were scored as 1; moderate lesions were scored as 2; severe lesions were scored as 3; very severe lesions were scored as 4*.

**Figure 8:**
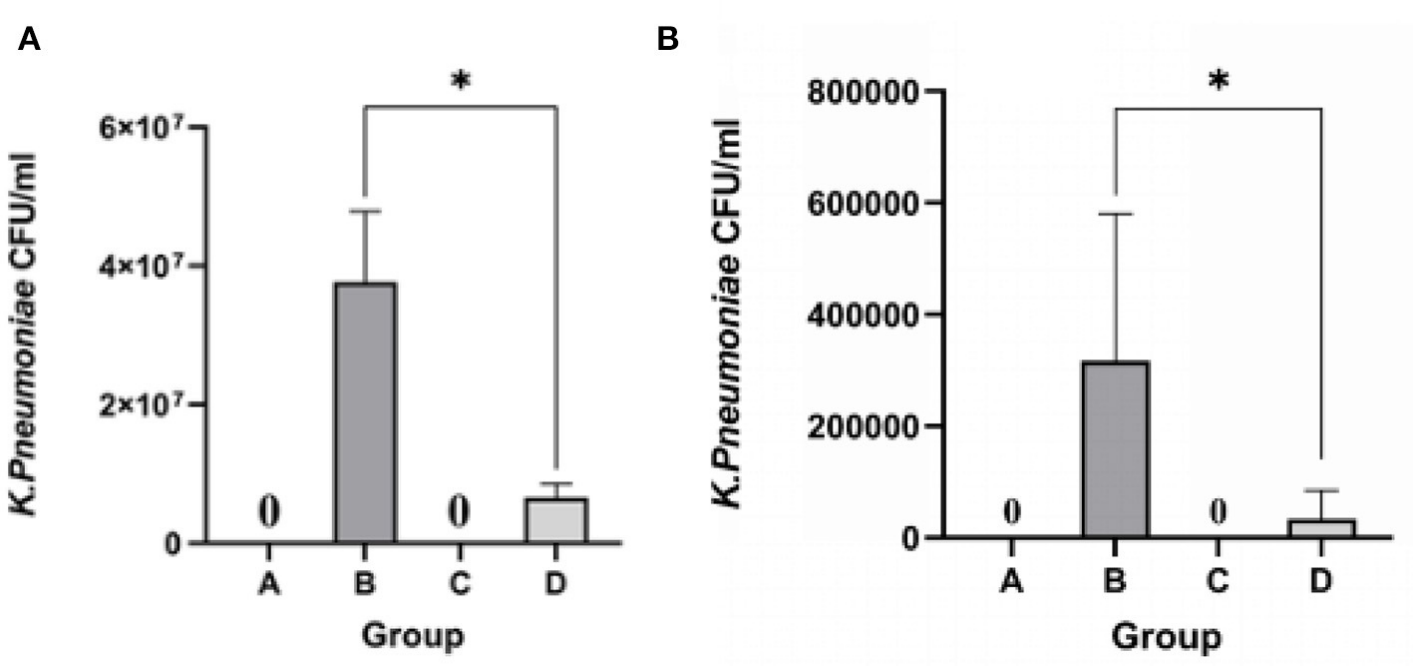
Count of bacterial load in lung and liver tissues of mice. **(A)** Bacterial load in lung tissues; **(B)** bacterial load in liver tissues; *p <0.05.

KP 18 was not detected in either lung or liver samples from the blank control and phage groups. The bacterial load in the lung of the bacterial group was 4.87 × 10^7^ CFU/mL and in the liver was 3.48 × 10^5^ CFU/mL. In the treatment group, 4 h after injecting phage B01, the bacterial load in the lungs decreased to 9.84 × 10^6^ CFU/mL, and the bacterial load in the liver also decreased (Figure 8). The bacterial load in the treatment group was significantly lower than that in the bacterial group.

Taken together, it is known that phage vB_Kpn_B01 can reduce the proliferation of *K. pneumoniae* KP 18 *in vivo*.

## Discussion

Bacteriophages have a high degree of host specificity, which can kill specific bacterial types. There will be no imbalance in the flora of the body to avoid secondary infections. Compared with antibiotic therapy, which is prone to drug residues and drug-resistant bacteria, phage therapy particularly highlights its advantages (26). First, if phage is used as a biological agent for biological control, its tolerance to physiochemical factors must be considered. In this study, *K. pneumoniae* phage vB_Kpn_B01 was isolated from the drinking trough of a dairy farm in Sichuan Province, and its biological properties and morphology were determined. Transmission electron microscopy revealed that it belongs to *the family Siphoviridae*. vB_Kpn_B01 had a wide range of adaptability to temperature and low tolerance to strong acids and alkalis. Obtaining the MOI (MOI = 0.01) and the burst size (42.5 PFU/infected cell) was used to lay the foundation for the preparation of phage biological agents. Due to the different effects of application conditions and the immune system, the life cycle of phages should be considered before they can be used as therapeutic agents (27, 28). MOI will determine the construction of phage cocktails and the effectiveness of phage reserves (29). In short, the biological properties of bacteriophages are a prerequisite for their ability to be used as therapeutic agents, and the theoretical basis for whether phages can be applied in production.

Genomics is an effective way to understand phage characteristics from a microscopic perspective. The genetic diversity and similarity of the genome are closely related to the lytic ability of the phage (30). From the CDS, vB_Kpn_B01 not only has related proteins that directly lyse the host but also have structural proteins that can participate in lysis. Endolysin (gp36) and Holin (gp37) are essential proteins for host lysis by bacteriophages (31). Two proteins, protein A1 (gp170) and protein A2 (gp173) are secondary structural proteins of bacteriophages. Protein A1 is essential for the production of infectious virus particles (32, 33). Protein A2 can inhibit MurA (34), an enzyme that catalyzes the first committed step in cell wall synthesis (35). The discovery of these proteins is beneficial for studying the phage lysis pathway. In addition, vB_Kpn_B01 was found to contain many tRNAs. Since viruses are highly dependent on host-translation mechanisms for protein synthesis, tRNA genes are occasionally identified in viruses (36–38). The presence of tRNA genes can reduce host dependence, expand the spectrum of the host, and improve its fitness (39, 40). These studies indicate that vB_Kpn_B01 is easier to preserve and may have a wide cleavage spectrum and are suitable for use as biological agents. Whole-genome sequence analysis showed that vB_Kpn_B01 genomes did not contain any known virulence, toxin, antibiotic resistance-encoding, or virulence regulator genes. Hence, vB_Kpn_B01 has higher safety for production (41, 42).

Several studies have already shown that phage therapy for the treatment of subclinical disease is effective in dairy cattle (43–45). However, most studies have focused on mastitis caused by *Staphylococcus aureus*. Meanwhile, to reduce the emergence of phage-resistant strains of *K. pneumoniae* during treatment, research has begun to explore the use of phage cocktails or combinations of phage therapy with antimicrobial agents (46, 47). This study explored the initial application of vB_Kpn_B01 *in vivo*. A mouse infection model was established by intraperitoneal injection of KP 18 (48). To further confirm that phage vB_Kpn_B01 can inhibit host bacterial multiplication and alleviate inflammation caused by *K. pneumoniae in vivo*, liver, and lung (49), the target organs mainly invaded by *K. pneumoniae* were selected to determine histopathological changes and bacterial load counts in organs. Histopathology of lungs and livers from mice infected with KP 18 showed significant alleviation of inflammation after phage treatment. Moreover, the bacterial load in these organs was more indicative of the inhibition of the reproduction of KP 18 *in vivo* after phage treatment, which is consistent with the results of previous trials on the treatment of *K. pneumoniae* (50, 51). However, in the test, it was found that the mice in the phage group (infected with vB_Kpn_B01 only) were cold and clustered together, while histopathological findings in this group also indicated mild infiltration of inflammatory cells. This may be because phages are foreign substances in the body and can stimulate the body to produce non-specific immunity, which, although helpful in clearing bacterial infections, may also allow the body to mount an inflammatory response (52–54).

In short, this assay demonstrated that bacteriophage vB_Kpn_B01 did have a therapeutic effect on mice infected with KP 18, which effectively alleviated bacterial infection *in vivo*; the result could once again prove that phage therapy is a viable alternative therapy for bacterial infectious diseases.

## Conclusion

In conclusion, we report on the biological properties of vB_Kpn_B01 with efficient growth characteristics and low tolerance to adverse environments. This phage belongs to the *Siphoviridae* family, *Sugarlandvirus*, and its features suggest that vB_Kpn_B01 has the potential to be used as a therapeutic agent. After the animal test, vB_Kpn_B01 showed significant inhibition of the KP 18 colonization in the lung and liver, effectively alleviating inflammation, demonstrating the extent of therapeutic efficacy, and having the potential to be developed into a clinical therapeutic agent for animal husbandry.

## Data Availability Statement

The datasets presented in this study can be found in online repositories. The names of the repository/repositories and accession number(s) can be found in the article/Supplementary Material.

## Ethics Statement

The animal study was reviewed and approved by Committee on Experimental Animal Management of the Sichuan Agricultural University (Approval No. SYXK2019-187).

## Author Contributions

ZL and SG conceived the project and designed the experiments. BL and GH performed the majority of the experiments. ZL wrote the manuscript. SC, YW, YL, ZY, and XY supervised the work and edited the final version of the manuscript. All authors have read and approved the final manuscript.

## Conflict of Interest

The authors declare that the research was conducted in the absence of any commercial or financial relationships that could be construed as a potential conflict of interest.

## Publisher's Note

All claims expressed in this article are solely those of the authors and do not necessarily represent those of their affiliated organizations, or those of the publisher, the editors and the reviewers. Any product that may be evaluated in this article, or claim that may be made by its manufacturer, is not guaranteed or endorsed by the publisher.
