# Supplementary material for: Isolation, Genomic Analysis, and Preliminary Application of a Bovine Klebsiella pneumoniae Bacteriophage vB_Kpn_B01
Source: Front Vet Sci. 2021 Sep 3;8:622049. doi: 10.3389/fvets.2021.622049 (PMC8446446; doi:10.3389/fvets.2021.622049)
Supplement: Supplementary file 1 [file Table_1.DOCX]

**Supplementary Table 1.** Genes with proposed functions in the genome of KP_LZD_B01

| Gene acccession | | Feature | Direction | Nucleotide position | | Length(bp) | Function |  |  |  |  |  |
| --- | --- | --- | --- | --- | --- | --- | --- | --- | --- | --- | --- | --- |
|  |  |  |  | start | end |  |  |  |  |  |  |  |
| gp1 |  | CDS | - | 174 | 341 | 168 | hypothetical proteins | | |  |  |  |
| gp2 |  | CDS | - | 1194 | 1541 | 348 | hypothetical proteins | | |  |  |  |
| gp3 |  | CDS | - | 1538 | 2062 | 525 | hypothetical proteins | | |  |  |  |
| gp4 |  | CDS | - | 2117 | 2611 | 495 | hypothetical protein | | |  |  |  |
| gp5 |  | CDS | - | 2677 | 3045 | 369 | hypothetical protein | | |  |  |  |
| gp6 |  | CDS | - | 3125 | 3643 | 519 | hypothetical protein | | |  |  |  |
| gp7 |  | CDS | - | 3716 | 4054 | 339 | hypothetical protein | | |  |  |  |
| gp8 |  | CDS | + | 4199 | 4312 | 114 | hypothetical protein | | |  |  |  |
| gp9 |  | CDS | + | 4312 | 4482 | 171 | hypothetical protein | | |  |  |  |
| gp10 |  | CDS | + | 4479 | 4622 | 144 | hypothetical protein | | |  |  |  |
| gp11 |  | CDS | - | 6313 | 7050 | 738 | hypothetical protein | | |  |  |  |
| gp12 |  | CDS | - | 7047 | 7484 | 438 | hypothetical protein | | |  |  |  |
| gp13 |  | CDS | - | 7533 | 7853 | 321 | hypothetical protein | | |  |  |  |
| gp14 |  | CDS | - | 7866 | 8375 | 510 | hypothetical protein | | |  |  |  |
| gp15 |  | CDS | - | 8388 | 8789 | 402 | hypothetical protein | | |  |  |  |
| gp16 |  | CDS | - | 8767 | 9243 | 477 | hypothetical protein | | |  |  |  |
| gp17 |  | CDS | - | 9243 | 9431 | 189 | hypothetical protein | | |  |  |  |
| gp18 |  | CDS | - | 9483 | 9770 | 288 | hypothetical protein | | |  |  |  |
| gp19 |  | CDS | - | 9781 | 10170 | 390 | hypothetical protein | | |  |  |  |
| gp20 |  | CDS | - | 10130 | 10597 | 468 | hypothetical protein | | |  |  |  |
| gp21 |  | CDS | - | 10581 | 10808 | 228 | hypothetical protein | | |  |  |  |
| gp22 |  | CDS | - | 10801 | 11142 | 342 | hypothetical protein | | |  |  |  |
| gp23 |  | CDS | - | 11139 | 11387 | 249 | hypothetical protein | | |  |  |  |
| gp24 |  | CDS | - | 11384 | 11665 | 282 | hypothetical protein | | |  |  |  |
| gp25 |  | CDS | - | 11665 | 11970 | 306 | hypothetical protein | | |  |  |  |
| gp26 |  | CDS | - | 12052 | 12480 | 429 | hypothetical protein | | |  |  |  |
| gp27 |  | CDS | - | 12544 | 12663 | 120 | hypothetical protein | | |  |  |  |
| gp28 |  | CDS | - | 12666 | 13034 | 369 | hypothetical protein | | |  |  |  |
| gp29 |  | CDS | - | 13043 | 13276 | 234 | hypothetical protein | | |  |  |  |
| gp30 |  | CDS | - | 13287 | 14117 | 831 | Serine/threonine-protein phosphatase | | | |  |  |
| gp31 |  | CDS | - | 14117 | 14467 | 351 | hypothetical protein | | |  |  |  |
| gp32 |  | CDS | - | 14468 | 14869 | 402 | hypothetical protein | | |  |  |  |
| gp33 |  | CDS | - | 14971 | 15255 | 285 | hypothetical protein | | |  |  |  |
| gp34 |  | CDS | - | 15248 | 15652 | 405 | hypothetical protein | | |  |  |  |
| gp35 |  | CDS | - | 15725 | 16141 | 417 | hypothetical protein | | |  |  |  |
| gp36 |  | CDS | - | 16202 | 16615 | 414 | L-alanyl-D-glutamate peptidase | | | |  |  |
| gp37 |  | CDS | - | 16612 | 17274 | 663 | Holin |  |  |  |  |  |
| gp38 |  | CDS | - | 17432 | 18043 | 612 | hypothetical protein | | |  |  |  |
| gp39 |  | CDS | - | 18053 | 18814 | 762 | Deoxynucleoside-5'-monophosphate kinase | | | | |  |
| gp40 |  | CDS | - | 19053 | 19514 | 462 | hypothetical protein | | |  |  |  |
| gp41 |  | CDS | - | 19480 | 20193 | 714 | hypothetical protein | | |  |  |  |
| gp42 |  | CDS | - | 20302 | 20694 | 393 | hypothetical protein | | |  |  |  |
| gp43 |  | CDS | - | 20814 | 21248 | 435 | hypothetical protein | | |  |  |  |
| gp44 |  | CDS | - | 21241 | 21513 | 273 | hypothetical protein | | |  |  |  |
| gp45 |  | CDS | - | 21506 | 21829 | 324 | hypothetical protein | | |  |  |  |
| gp46 |  | CDS | - | 21876 | 22313 | 438 | hypothetical protein | | |  |  |  |
| gp47 |  | CDS | - | 22315 | 22524 | 210 | hypothetical protein | | |  |  |  |
| gp48 |  | CDS | - | 22521 | 22826 | 306 | hypothetical protein | | |  |  |  |
| gp49 |  | CDS | - | 22816 | 23082 | 267 | hypothetical protein | | |  |  |  |
| gp50 |  | CDS | - | 23051 | 23413 | 363 | hypothetical protein | | |  |  |  |
| gp51 |  | CDS | - | 23406 | 23633 | 228 | hypothetical protein | | |  |  |  |
| gp52 |  | CDS | - | 23620 | 24162 | 543 | hypothetical protein | | |  |  |  |
| gp53 |  | CDS | - | 24162 | 24434 | 273 | hypothetical protein | | |  |  |  |
| gp54 |  | CDS | - | 24438 | 24635 | 198 | hypothetical protein | | |  |  |  |
| gp55 |  | CDS | - | 24748 | 24924 | 177 | hypothetical protein | | |  |  |  |
| gp56 |  | CDS | - | 24926 | 25306 | 381 | hypothetical protein | | |  |  |  |
| gp57 |  | CDS | - | 25297 | 25653 | 357 | hypothetical protein | | |  |  |  |
| gp58 |  | tRNA | - | 25670 | 25746 | 77 | tRNA-Trp(cca) | |  |  |  |  |
| gp59 |  | CDS | - | 25751 | 25942 | 192 | hypothetical protein | | |  |  |  |
| gp60 |  | CDS | - | 26061 | 26162 | 102 | hypothetical protein | | |  |  |  |
| gp61 |  | CDS | - | 26330 | 26728 | 399 | hypothetical protein | | |  |  |  |
| gp62 |  | tRNA | - | 26744 | 26817 | 74 | tRNA-Thr(tgt) | |  |  |  |  |
| gp63 |  | tRNA | - | 26823 | 26899 | 77 | tRNA-Pro(tgg) | |  |  |  |  |
| gp64 |  | tRNA | - | 27104 | 27181 | 78 | tRNA-Leu(taa) | |  |  |  |  |
| gp65 |  | CDS | - | 27183 | 27332 | 150 | hypothetical protein | | |  |  |  |
| gp66 |  | CDS | - | 27332 | 27670 | 339 | hypothetical protein | | |  |  |  |
| gp67 |  | CDS | - | 27673 | 27867 | 195 | hypothetical protein | | |  |  |  |
| gp68 |  | tRNA | - | 28312 | 28389 | 78 | tRNA-Phe(gaa) | |  |  |  |  |
| gp69 |  | tRNA | - | 28565 | 28640 | 76 | tRNA-Lys(ttt) | |  |  |  |  |
| gp70 |  | tRNA | - | 28824 | 28897 | 74 | tRNA-Cys(gca) | |  |  |  |  |
| gp71 |  | tRNA | - | 28978 | 29053 | 76 | tRNA-Asn(gtt) | |  |  |  |  |
| gp72 |  | tRNA | - | 29163 | 29249 | 87 | tRNA-Tyr(gta) | |  |  |  |  |
| gp73 |  | tRNA | - | 29389 | 29465 | 77 | tRNA-Asp(gtc) | |  |  |  |  |
| gp74 |  | CDS | - | 29474 | 29587 | 114 | hypothetical protein | | |  |  |  |
| gp75 |  | CDS | - | 29659 | 29874 | 216 | hypothetical protein | | |  |  |  |
| gp76 |  | CDS | - | 29874 | 30251 | 378 | hypothetical protein | | |  |  |  |
| gp77 |  | CDS | - | 30272 | 30544 | 273 | hypothetical protein | | |  |  |  |
| gp78 |  | tRNA | - | 30840 | 30915 | 76 | tRNA-Val(tac) | |  |  |  |  |
| gp79 |  | tRNA | - | 30919 | 30995 | 77 | tRNA-Met(cat) | |  |  |  |  |
| gp80 |  | tRNA | - | 31153 | 31228 | 76 | tRNA-Ile(gat) | |  |  |  |  |
| gp81 |  | CDS | - | 31230 | 31388 | 159 | hypothetical protein | | |  |  |  |
| gp82 |  | CDS | - | 31396 | 31587 | 192 | hypothetical protein | | |  |  |  |
| gp83 |  | tRNA | - | 31604 | 31680 | 77 | tRNA-Met(cat) | |  |  |  |  |
| gp84 |  | tRNA | - | 31683 | 31758 | 76 | tRNA-Gln(ctg) | |  |  |  |  |
| gp85 |  | tRNA | - | 31888 | 31962 | 75 | tRNA-Arg(acg) | |  |  |  |  |
| gp86 |  | tRNA | - | 31970 | 32046 | 77 | tRNA-His(gtg) | |  |  |  |  |
| gp87 |  | tRNA | - | 32429 | 32515 | 87 | tRNA-Ser(gct) | |  |  |  |  |
| gp88 |  | tRNA | - | 32523 | 32607 | 85 | tRNA-Ser(tga) | |  |  |  |  |
| gp89 |  | tRNA | - | 32719 | 32805 | 87 | tRNA-Leu(tag) | |  |  |  |  |
| gp90 |  | tRNA | - | 32813 | 32891 | 79 | tRNA-Ala(tgc) | |  |  |  |  |
| gp91 |  | tRNA | - | 33175 | 33251 | 77 | tRNA-Glu(ttc) | |  |  |  |  |
| gp92 |  | tRNA | - | 33262 | 33337 | 76 | tRNA-Gln(ttg) | |  |  |  |  |
| gp93 |  | CDS | - | 33432 | 33926 | 495 | hypothetical protein | | |  |  |  |
| gp94 |  | CDS | - | 33937 | 34146 | 210 | hypothetical protein | | |  |  |  |
| gp95 |  | CDS | - | 34146 | 34343 | 198 | hypothetical protein | | |  |  |  |
| gp96 |  | tRNA | - | 34365 | 34442 | 78 | tRNA-Met(cat) | |  |  |  |  |
| gp97 |  | CDS | - | 34473 | 34766 | 294 | hypothetical protein | | |  |  |  |
| gp98 |  | tRNA | - | 34918 | 34993 | 76 | tRNA-Arg(tct) | |  |  |  |  |
| gp99 |  | CDS | - | 34995 | 35171 | 177 | hypothetical protein | | |  |  |  |
| gp100 |  | CDS | - | 35174 | 35500 | 327 | hypothetical protein | | |  |  |  |
| gp101 |  | CDS | - | 36781 | 37242 | 462 | hypothetical protein | | |  |  |  |
| gp102 |  | CDS | - | 37307 | 38311 | 1005 | hypothetical protein | | |  |  |  |
| gp103 |  | CDS | - | 38311 | 38721 | 411 | hypothetical protein | | |  |  |  |
| gp104 |  | CDS | - | 38851 | 39135 | 285 | hypothetical protein | | |  |  |  |
| gp105 |  | CDS | - | 39125 | 39286 | 162 | hypothetical protein | | |  |  |  |
| gp106 |  | CDS | - | 39286 | 39768 | 483 | hypothetical protein | | |  |  |  |
| gp107 |  | CDS | - | 39768 | 40043 | 276 | hypothetical protein | | |  |  |  |
| gp108 |  | CDS | - | 40141 | 40374 | 234 | hypothetical protein | | |  |  |  |
| gp109 |  | CDS | - | 40381 | 41235 | 855 | Thymidylate synthase | | |  |  |  |
| gp110 |  | CDS | - | 41232 | 41795 | 564 | hypothetical protein | | |  |  |  |
| gp111 |  | CDS | - | 41795 | 43081 | 1287 | hypothetical protein | | |  |  |  |
| gp112 |  | CDS | - | 43178 | 45502 | 2325 | Ribonucleoside-diphosphate reductase large subunit | | | | | |
| gp113 |  | CDS | - | 45631 | 45840 | 210 | hypothetical protein | | |  |  |  |
| gp114 |  | CDS | - | 45855 | 45968 | 114 | hypothetical protein | | |  |  |  |
| gp115 |  | CDS | - | 45970 | 46719 | 750 | hypothetical protein | | |  |  |  |
| gp116 |  | CDS | + | 47030 | 48859 | 1830 | Anaerobic ribonucleoside-triphosphate reductase | | | | |  |
| gp117 |  | CDS | + | 48920 | 49129 | 210 | hypothetical protein | | |  |  |  |
| gp118 |  | CDS | + | 49131 | 49964 | 834 | hypothetical protein | | |  |  |  |
| gp119 |  | CDS | + | 49948 | 50112 | 165 | hypothetical protein | | |  |  |  |
| gp120 |  | CDS | + | 50099 | 50500 | 402 | hypothetical protein | | |  |  |  |
| gp121 |  | CDS | + | 50497 | 50892 | 396 | hypothetical protein | | |  |  |  |
| gp122 |  | CDS | + | 51491 | 54280 | 2790 | Putative replication origin binding protein | | | | |  |
| gp123 |  | CDS | + | 54267 | 54551 | 285 | hypothetical protein | | |  |  |  |
| gp124 |  | CDS | + | 54584 | 55279 | 696 | hypothetical protein | | |  |  |  |
| gp125 |  | CDS | + | 55272 | 55490 | 219 | hypothetical protein | | |  |  |  |
| gp126 |  | CDS | + | 55565 | 55969 | 405 | hypothetical protein | | |  |  |  |
| gp127 |  | CDS | + | 55982 | 56278 | 297 | hypothetical protein | | |  |  |  |
| gp128 |  | CDS | + | 56329 | 56637 | 309 | hypothetical protein | | |  |  |  |
| gp129 |  | CDS | + | 56721 | 56858 | 138 | hypothetical protein | | |  |  |  |
| gp130 |  | CDS | + | 56839 | 57114 | 276 | hypothetical protein | | |  |  |  |
| gp131 |  | CDS | + | 57114 | 58076 | 963 | DNA ligase | |  |  |  |  |
| gp132 |  | CDS | + | 58276 | 59025 | 750 | hypothetical protein | | |  |  |  |
| gp133 |  | CDS | + | 59028 | 59786 | 759 | Putative transcription factor D5 | | | |  |  |
| gp134 |  | CDS | + | 59871 | 61349 | 1479 | hypothetical protein | | |  |  |  |
| gp135 |  | CDS | + | 61346 | 62230 | 885 | hypothetical protein | | |  |  |  |
| gp136 |  | CDS | + | 62298 | 64868 | 2571 | DNA polymerase | |  |  |  |  |
| gp137 |  | CDS | + | 65355 | 66713 | 1359 | putative helicase D10 | | |  |  |  |
| gp138 |  | CDS | + | 66916 | 67269 | 354 | hypothetical protein | | |  |  |  |
| gp139 |  | CDS | + | 67272 | 68045 | 774 | putative ssDNA-binding protein | | | |  |  |
| gp140 |  | CDS | + | 68085 | 69077 | 993 | putative exonuclease subunit 1 | | | |  |  |
| gp141 |  | CDS | + | 69061 | 70896 | 1836 | putative exonuclease subunit 2 | | | |  |  |
| gp142 |  | CDS | + | 70896 | 71378 | 483 | Protein D14 | |  |  |  |  |
| gp143 |  | CDS | + | 71375 | 72253 | 879 | Flap endonuclease | |  |  |  |  |
| gp144 |  | CDS | + | 72253 | 72696 | 444 | Deoxyuridine 5'-triphosphate nucleotidohydrolase | | | | |  |
| gp145 |  | CDS | - | 72738 | 74780 | 2043 | hypothetical protein | | |  |  |  |
| gp146 |  | CDS | - | 74780 | 75199 | 420 | L-shaped tail fiber protein p132 | | | |  |  |
| gp147 |  | CDS | - | 75203 | 85600 | 10398 | hypothetical protein | | |  |  |  |
| gp148 |  | CDS | - | 85628 | 88474 | 2847 | putative baseplate hub protein | | | |  |  |
| gp149 |  | CDS | - | 88471 | 89085 | 615 | Distal tail protein | |  |  |  |  |
| gp150 |  | CDS | - | 89200 | 94278 | 5079 | hypothetical protein | | |  |  |  |
| gp151 |  | CDS | - | 94363 | 94689 | 327 | hypothetical protein | | |  |  |  |
| gp152 |  | CDS | - | 94763 | 95185 | 423 | hypothetical protein | | |  |  |  |
| gp153 |  | CDS | - | 95189 | 96076 | 888 | Minor tail protein | |  |  |  |  |
| gp154 |  | CDS | - | 96089 | 97219 | 1131 | Tail tube protein | |  |  |  |  |
| gp155 |  | CDS | - | 97243 | 97728 | 486 | Tail tube terminator protein | | |  |  |  |
| gp156 |  | CDS | - | 97728 | 98489 | 762 | Tail completion protein | | |  |  |  |
| gp157 |  | CDS | - | 98489 | 98992 | 504 | hypothetical protein | | |  |  |  |
| gp158 |  | CDS | - | 99060 | 100442 | 1383 | Major capsid protein | | |  |  |  |
| gp159 |  | CDS | - | 100456 | 101052 | 597 | Prohead protease | |  |  |  |  |
| gp160 |  | CDS | - | 101249 | 101719 | 471 | hypothetical protein | | |  |  |  |
| gp161 |  | CDS | - | 101716 | 102927 | 1212 | Portal protein | |  |  |  |  |
| gp162 |  | CDS | + | 103318 | 103518 | 201 | hypothetical protein | | |  |  |  |
| gp163 |  | CDS | - | 103601 | 104917 | 1317 | Terminase, large subunit | | |  |  |  |
| gp164 |  | CDS | - | 104917 | 105390 | 474 | putative terminase, small subunit | | | |  |  |
| gp165 |  | CDS | - | 105401 | 107377 | 1977 | Receptor-binding protein | | |  |  |  |
| gp166 |  | CDS | + | 107491 | 107715 | 225 | hypothetical protein | | |  |  |  |
| gp167 |  | CDS | + | 107719 | 108174 | 456 | hypothetical protein | | |  |  |  |
| gp168 |  | CDS | - | 108339 | 109055 | 717 | 5'-deoxynucleotidase | | |  |  |  |
| gp169 |  | CDS | - | 109128 | 109580 | 453 | hypothetical protein | | |  |  |  |
| gp170 |  | CDS | - | 109644 | 111326 | 1683 | Protein A1 | |  |  |  |  |
| gp171 |  | CDS | - | 111410 | 111637 | 228 | hypothetical protein | | |  |  |  |
| gp172 |  | CDS | - | 111634 | 111903 | 270 | hypothetical protein | | |  |  |  |
| gp173 |  | CDS | - | 111971 | 112375 | 405 | Protein A2 | |  |  |  |  |
| gp174 |  | CDS | - | 112715 | 112975 | 261 | hypothetical protein | | |  |  |  |
